# Longitudinal Changes in the Digitized Archimedes Spiral Drawing Test Following iTBS of the Left DLPFC in Parkinson’s Disease: An Exploratory Feasibility Study

**DOI:** 10.3390/jcm15197754

**Published:** 2026-10-08

**Authors:** Alexander Zakharov, Mariya Sergeeva, Yuliya Komarova, Natalia Romanchuk, Vladimir Khorev, Igor Shirolapov

**Affiliations:** Research Institute of Neurosciences, Samara State Medical University, 443099 Samara, Russia; a.v.zaharov@samsmu.ru (A.Z.); m.s.sergeeva@samsmu.ru (M.S.); ju.s.komarova@samsmu.ru (Y.K.); n.p.romanchuk@samsmu.ru (N.R.);

**Keywords:** Parkinson’s disease, Digitized Archimedes Spiral Drawing Test (DAST), intermittent theta-burst stimulation (iTBS), dorsolateral prefrontal cortex (DLPFC), tremor

## Abstract

**Background:** The Digitized Archimedes Spiral Drawing Test (DAST) provides objective assessment of fine motor function, but its utility as a biomarker for neuromodulatory interventions in Parkinson’s disease (PD) remains underexplored. **Objective:** To explore longitudinal changes in a comprehensive set of DAST parameters following a course of intermittent theta-burst stimulation (iTBS) of the left dorsolateral prefrontal cortex (DLPFC) in PD patients as a feasibility study. **Methods:** Twenty-seven participants (17 PD patients, 10 healthy controls) underwent DAST testing at baseline and at 1.5 and 6 months; the immediate post-course assessment was performed only in the PD group, whereas the controls received a single sham session. Forty kinematic, temporal, spatial and tremor-related metrics were extracted. Clinical assessment was performed using MDS-UPDRS. Linear mixed models, ANCOVA, and Friedman tests were applied for statistical analysis. **Results:** Temporal and kinematic parameters showed modest changes over the study period, including mean velocity (*p* = 0.0437), trial duration (*p* = 0.0437), and mean number of movements (*p* = 0.0485). In contrast, spatial parameters (total error and length-normalized error) showed no significant change over the study period but maintained stable between-group differences across all stages (*p* = 0.0251 and *p* = 0.0123 at 6 months). Age and sex significantly influenced several DAST metrics: age affected mean error for spiral types 2, 3, and 4 (*p* = 0.008–0.047), while sex influenced dominant tremor frequency, tremor power ratio (*p* = 0.005–0.007), and mean error for spiral types 3 and 4 (*p* = 0.023–0.039). The DAST showed limited ability to differentiate adjacent PD stages: only mean velocity of spiral type 2 differed between Hoehn and Yahr stages 2 and 3 at baseline (*p* = 0.0176), with no significant differences between subgroups at 6 months. **Conclusions:** The changes over the study period were observed in kinematic and temporal DAST measures, but not in spatial parameters. These preliminary findings may suggest a possible dissociation between parameters predominantly reflecting motor planning and those reflecting trajectory execution. It may be tentatively hypothesized that DLPFC stimulation primarily modulates motor planning processes and movement execution strategies, whereas trajectory implementation and spatial coordination appear to be mediated by other cortico-subcortical structures. DAST shows potential as an objective tool for monitoring motor changes in PD; nevertheless, its responsiveness should be further evaluated in larger studies.

## 1. Introduction

Parkinson’s disease (PD) is a heterogeneous and progressive neurodegenerative disorder characterized by a broad spectrum of motor and non-motor symptoms. In clinical practice, the diagnosis of PD continues to rely predominantly on the assessment of motor disturbances, with bradykinesia and rigidity often manifesting already at early stages [1,2,3]. These impairments significantly affect handwriting and graphomotor function in patients, which is why micrographia is considered one of the early diagnostic signs of PD [4]. At the same time, handwriting characteristics may be influenced by educational level, language skills, and other cognitive factors, which limits its diagnostic specificity [5,6]. In this regard, the execution of graphic tasks, particularly drawing the Archimedes spiral, has proven to be a non-invasive and informative method for assessing fine motor skills, less sensitive to linguistic and cultural influences. To date, the use of modern sensory digital devices for such tasks provides accurate recording of stylus spatial position, temporal characteristics, pressure force, stylus tilt angle, and in-air movement parameters. This substantially expands the possibilities for quantitative assessment of the kinematic, dynamic, and spatial characteristics of the drawing [7,8].

Analysis of the spiral drawing test is based on digital processing of the obtained data, including temporal parameters, X and Y coordinates, as well as pressure indicators on the touchscreen. This approach allows the calculation of numerous quantitative indices reflecting various aspects of motor dysfunction. The kinematics of the spiral drawing reveal features such as tremor amplitude, degree of bradykinesia and dyskinesia, and also enable differentiation between distal and proximal tremors [9]. The computed indices correlate with clinical rating scales in PD, essential tremor, and other movement disorders, and also capture physiological indicators insufficiently represented in several clinical scales, such as micrographia [10,11]. Moreover, the Digitized Archimedes Spiral drawing test (DAST) can be considered a sensitive biomarker of cognitive deficit, reflecting impairments in executive and motor control [12,13].

In recent years, compelling evidence has accumulated on the potential of DAST as a promising tool for detecting treatment-related changes in motor function in PD and essential tremor. In particular, parameters such as drawing velocity and tremor intensity consistently reflect improvement following various therapeutic interventions, including levodopa administration, MRI-guided focused ultrasound thalamotomy, or deep brain stimulation [8,10]. However, the number of studies evaluating the sensitivity of the method to clinically significant changes remains limited. This underscores the need for further investigation of DAST in a broader clinical context, as well as for justification of its use as a reliable outcome measure for monitoring therapeutic efficacy. Furthermore, considerable methodological discrepancies persist regarding test administration, device selection, task types, and data analysis approaches, complicating standardization and cross-study comparisons [14]. It should also be emphasized that DAST measures are influenced by age, sex, and the hand used, necessitating consideration of these factors when interpreting results [15].

The dorsolateral prefrontal cortex (DLPFC) is a key hub of frontal networks responsible for executive functions, cognitive motor control, and integration of motor and sensory information. The DLPFC is involved in planning voluntary movements, regulating the precision and consistency of motor programs, as well as in suppressing uncontrolled motor patterns. Current understanding of PD pathophysiology extends beyond the classical concept of predominant damage to the extrapyramidal system: emerging functional neuroanatomical data indicate involvement of various brain regions and structures in the pathological process, including the DLPFC [16,17]. In particular, alterations in the frontoparietal neuronal network have been established, including hypometabolism and thinning of frontostriatal projection tracts, which correlate with the severity of impairments in cognitive control, planning, and voluntary attention in PD patients. Moreover, the DLPFC plays an important role in the development of depressive symptoms and sleep disturbances, which frequently present as comorbid manifestations in PD patients and may exacerbate cognitive and motor dysfunctions [18,19,20,21,22].

Non-invasive brain stimulation (NIBS), including repetitive transcranial magnetic stimulation (rTMS) and transcranial direct current stimulation (tDCS), can modulate dopamine release in the caudate nucleus and putamen, as well as in their cortico-striatal projections. With respect to motor symptoms in PD, recent meta-analyses indicate that rTMS exerts a small to moderate positive effect on overall motor function and functional mobility (gait speed and the Timed Up and Go test), whereas effects on static balance remain negligible [23,24]. In turn, theta-burst stimulation (TBS), a variant of rTMS, offers advantages of short session duration and low intensity, and its application over the primary motor cortex (M1) and DLPFC has demonstrated additional therapeutic effects on depression in PD patients [25]. At the same time, DLPFC stimulation may exert a specific influence on cognitive motor control and strategies for performing graphomotor tasks, making DAST a particularly sensitive tool for assessing such effects.

Performing a spiral drawing requires coordination of voluntary movements, integration of sensorimotor feedback, and precise control of stylus trajectory. In this regard, the DAST can be considered a sensitive instrument for indirect assessment of DLPFC functional state and its interaction with motor neuronal networks [11]. Studies on DAST validation have demonstrated high correlations between digital measures and clinical rating scales, as well as objective monitoring methods, and have confirmed the reliability of the method in repeated measurements [26,27]. Composite indices have been developed that demonstrate stronger correlations with PD severity than each individual test parameter, although their ability to distinguish between adjacent stages of the disease remains limited [9]. All of this underscores the need for further investigation of both isolated and integrative DAST parameters in the context of neuromodulatory interventions, as well as their sensitivity to therapy-induced changes.

The aim of this exploratory feasibility study was to assess longitudinal changes in a comprehensive set of DAST parameters following a course of intermittent theta-burst stimulation (iTBS) of the left DLPFC in patients with PD. Within the framework of this aim, the following objectives were addressed: first, to explore whether kinematic, temporal, and spatial DAST measures show differential responsiveness to the iTBS course; second, to assess the temporal stability of any observed changes over the follow-up period; and third, to compare the observed changes with disease severity and demographic factors (age, sex). In contrast to previous studies, we aimed not only to detect DAST changes following the iTBS course but also to explore the potential of DAST as a candidate biomarker and to identify which classes of parameters may be most informative for monitoring therapeutic outcomes in this context.

## 2. Materials and Methods

### 2.1. Study Population and Design

The study was conducted in accordance with the principles of the Declaration of Helsinki and was approved by the Local Ethics Committee of Samara State Medical University (Protocol No. 278 dated 30 January 2024). All participants provided written informed consent prior to inclusion in the study. The study design involved three temporal assessment stages: baseline, 1.5 months, and 6 months after the first visit. At each stage, all subjects performed the DAST. In patients with PD, clinical testing was additionally performed using the Unified Parkinson’s Disease Rating Scale of the Movement Disorder Society (MDS-UPDRS), Part III.

The specific patient recruitment period: from 1 March 2024 to 31 December 2024. The study included 27 individuals aged 65.44 ± 6.17 years with no contraindications to TMS. Given the exploratory design, the present sample was recruited on a feasibility basis and should be regarded as a pilot cohort without prospective power justification. Two groups were formed. The experimental group (ExpG) comprised 17 patients with a verified diagnosis of Parkinson’s disease (PD) according to the MDS criteria. The control group (CG) comprised 10 healthy volunteers of the same age group, with no history of neurological disorders, no complaints of resting tremor, no use of medications that could induce tremor, and no clinically significant fine motor deficits. Exclusion criteria for both groups included clinically confirmed or self-reported musculoskeletal injuries of the upper extremities, other neurological or neuromuscular diseases, excessive levodopa dosage causing dyskinesias, as well as pronounced cognitive impairments that prevented compliance with the instructions. Demographic and clinical characteristics of the participants are presented in Table 1.

### 2.2. TMS Protocol

In the ExpG, a course of iTBS of the left DLPFC was administered at the baseline stage as 10 daily sessions. During TMS, the participant was seated comfortably with head fixation using a vacuum cushion. Prior to the course, the resting motor threshold (RMT) was determined using single biphasic pulses delivered through a figure-of-eight CB60 coil (MagVenture, Farum, Denmark). The hotspot was defined as the cortical representation of the right abductor pollicis brevis (APB) muscle. RMT corresponded to the minimum stimulation intensity required to elicit 5 out of 10 motor evoked potentials (MEPs) with an amplitude of ≥50 μV in the relaxed APB [28]. Surface EMG was recorded with bipolar electrodes placed over the muscle belly distally and on the forearm. iTBS itself was delivered using a CoolB65 coil of the same configuration, connected to the same MagPro X100 stimulator (MagVenture). The left DLPFC was localized using neuronavigation according to the Fitzgerald protocol [29]. Stimulation intensity was 100% of RMT. The iTBS protocol [30] consisted of 2 s trains (3 pulses at 50 Hz repeated at 5 Hz) with an inter-train interval of 10 s over a total duration of 3 min (600 pulses in total). Before and after each session, discomfort (0–10 VAS) and alertness (0–10 numerical scale) were assessed. In the CG, sham TMS was applied once using a sham coil that was externally identical but generated no magnetic field (the healthy participants served as a reference group for DAST parameters rather than as a placebo control for the iTBS course). Given the absence of a sham-controlled PD group, the observed changes should be regarded as exploratory and hypothesis-generating.

### 2.3. Spiral Drawing Task and Data Acquisition

Assessment of graphomotor function was performed using a hardware-software system based on a Samsung Galaxy Tab S7 FE tablet (12.4-inch screen diagonal, resolution 2560 × 1600 pixels, sampling rate 120 Hz, Android 14, One UI 6.0) (Samsung Electronics Co., Ltd., Suwon, Republic of Korea). A stylus with pressure-sensitivity support (4096 levels) was used for the task. All recordings were made using a dedicated NeuroStim program V.1.4.4 (Neurosoft, Ivanovo, Russia), which synchronized temporal, spatial, and dynamic data. PD participants were assessed in the medication “on” state, and the time interval from the most recent dopaminergic dose to each DAST assessment was maintained as consistent as possible across visits, although it was not strictly standardized.

**Figure 1:**
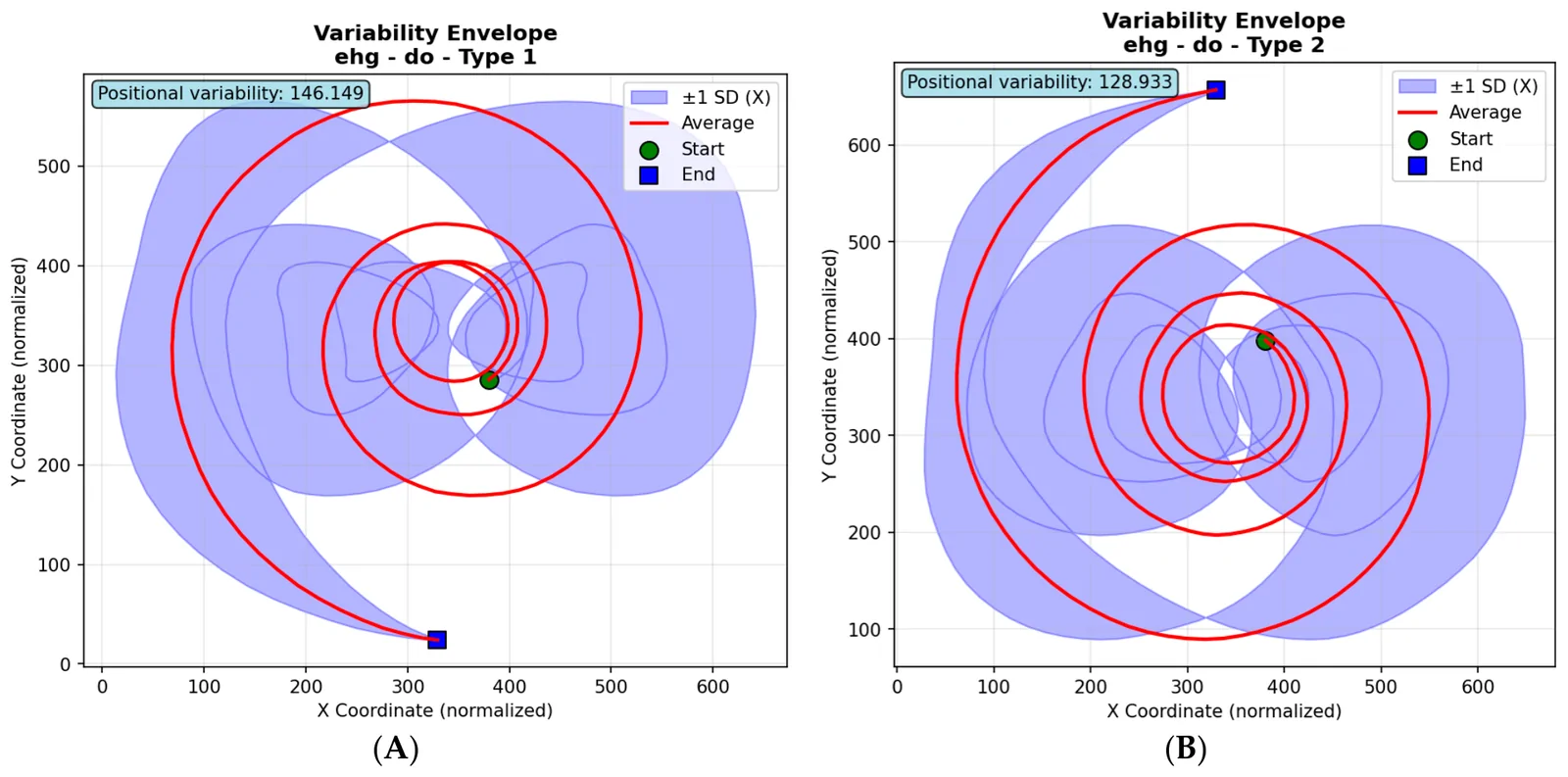

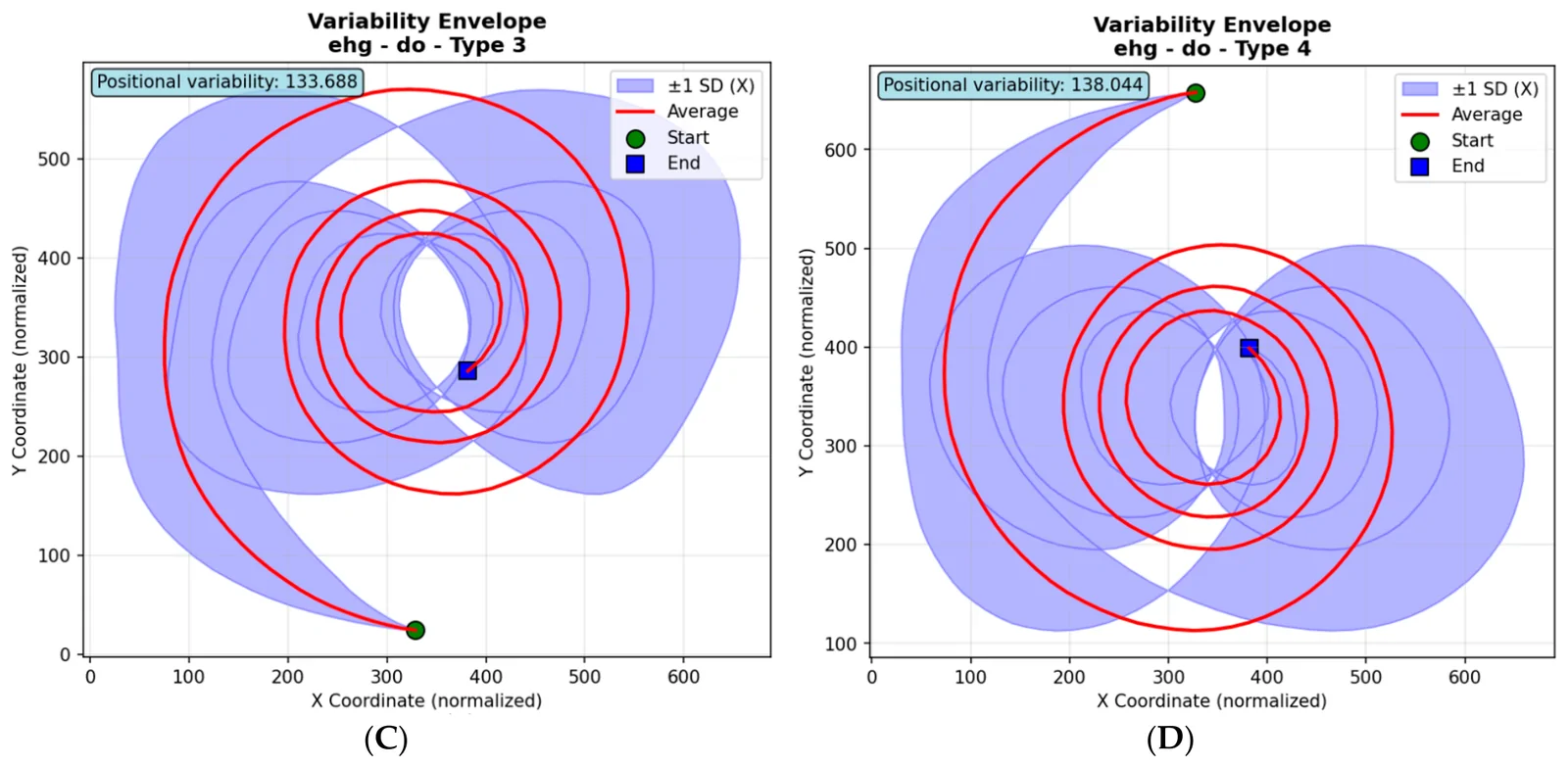
Schematic representation of the four spiral types that differ in starting point and drawing direction. (**A**)—starting point inside, counter-clockwise direction (spiral type 1); (**B**)—starting point inside, clockwise direction (spiral type 2); (**C**)—starting point outside, clockwise direction (spiral type 3); (**D**)—starting point outside, counter-clockwise direction (spiral type 4).

While seated at a table, the participant received the following instruction: Trace the presented spiral template as accurately and as quickly as possible, without lifting the stylus from the screen. The hand and wrist were not allowed to rest on the screen surface to minimize the influence of static friction on movement kinematics. The dominant hand (right for all subjects) was used. Four types of fixed-size spirals were presented, differing in the position of the starting point (inside or outside) and the drawing direction (clockwise or counter-clockwise), as shown in Figure 1. Each type was reproduced 6 times in a randomized order; the total number of attempts per stage was 24. The template parameters were as follows: 5 turns, outer radius 320 pixels, inner radius 67 pixels, diameter increment step 63 pixels, line thickness 7 pixels (equivalent to a pencil trace on paper). Participants performed one practice trial prior to testing to familiarize themselves with the task. In the ExpG, DAST testing at the first stage was performed twice: before the start of the iTBS course (baseline) and immediately after the completion of the last stimulation session, which allowed assessment of the immediate intervention effect. At the second and third stages, testing was performed once. DAST administration was performed by medical professionals (physicians); the data processing and metric computation were fully automated. To minimize subjective bias at the analysis stage, the investigators were blinded to participant group and time point.

### 2.4. Data Processing and Computed Measures

Primary data were saved in JSON format and contained participant metadata, event timestamps, stylus coordinates (X, Y), pressure (Z), and screen contact markers (pen-down/pen-up). Processing was performed in the Python environment (version 3.13).

For each trial, segmentation was performed by touch episodes, and the following conditional classes of measures were extracted from the time series: Spatial—radial error (deviation from the reference trajectory), total path length, spiral area, coefficient of variation in loop width; Temporal—total trial duration, number of movements (segments between pen-down and pen-up), duration of individual segments; Kinematic—instantaneous and mean velocity, standard deviation of velocity, coefficient of variation in velocity, jerk, spectral arc length (smoothness); Tremor—dominant tremor frequency (based on spectral analysis), ratio of power in the tremor frequency band to total signal power; Dynamic—mean and variability of stylus pressure, press frequency; Integrative—total error (sum of absolute deviations over all points), length-normalized error, approximate entropy. For each subject, mean values over 6 repetitions were calculated for each spiral type; for each group, group means and measures of variability were calculated. The complete list comprised 40 computed metrics. For each measure, the coefficient of variation (CV = SD/M × 100%) was additionally calculated, reflecting inter-trial variability within a single session.

### 2.5. Statistical Analysis

Statistical processing was performed using IBM SPSS Statistics (version 27) and R (version 4.2.1) software packages. The significance level was set at 0.05; Bonferroni correction was applied to post-hoc pairwise comparisons within the Friedman tests and to correlation analyses. The normality of the distribution of quantitative variables was assessed using the Shapiro–Wilk test both for raw data and for paired differences; a *p*-value > 0.05 was considered to indicate a normal distribution. Between-group comparisons at individual stages were performed using analysis of covariance (ANCOVA) with age and sex as covariates when the data were normally distributed (with calculation of the partial eta-squared coefficient, η^2^), or the Mann–Whitney test when normality was violated (with calculation of the rank-biserial correlation). Longitudinal analysis within the experimental group across four time points (baseline, after the iTBS course, at 1.5 months, and at 6 months) was conducted using the non-parametric Friedman test; when the result was significant, post-hoc pairwise comparisons were performed using the Wilcoxon test with Bonferroni correction. For pairwise comparisons of two time points, the paired *t*-test (when normality was met) or the Wilcoxon test was used. Stratification by disease stage (Hoehn and Yahr stages 2 and 3) was performed at each stage using Student’s *t*-test, Welch’s *t*-test (when variances were unequal), or the Mann–Whitney test with calculation of the effect size (Cohen’s d or rank-biserial correlation). To identify the combined influence of group, time point, age, and sex on DAST parameters, a linear mixed model was built with the structure: intercept + age + sex + group + time point + group × time point, with subject included as a random effect (random intercept); interactions were assessed using the likelihood ratio test. The model included the baseline, 1.5-month, and 6-month time points; the immediate post-treatment measurement, available only in the PD group, was not entered into the model to preserve a balanced design. Time was coded with baseline as the reference category. Correlation analysis between DAST parameters and clinical scales (MDS-UPDRS III) was performed using Spearman’s rank correlation coefficient. The between-group and between-stage comparisons were exploratory in nature, given the feasibility design of the study, and were reported with corresponding effect sizes (partial eta-squared for ANCOVA, rank-biserial correlation for Mann–Whitney tests, Cohen’s d for *t*-tests, and Kendall’s W for Friedman tests). All *p*-values reported are unadjusted unless explicitly stated otherwise.

## 3. Results

### 3.1. Effects of Age, Sex and Study Stage on DAST Parameters

Analysis of the linear mixed model revealed statistically significant effects of age, sex, and group affiliation on individual DAST measures. No significant group × time interaction was found for the principal DAST outcomes, including total error, length-normalized error, mean velocity, trial duration, mean number of movements, and mean duration for spiral type 2 (interaction *p* > 0.05 in all cases). In the adjusted model accounting for age and sex, the following parameters were significant: total path length (*p* = 0.029), ratio of tremor frequency power to total power (*p* = 0.017), total error (mismatch between the drawn spiral and the reference trajectory) (*p* = 0.028), and length-normalized error (*p* = 0.030). Total spiral area showed a trend that did not reach the significance threshold (*p* = 0.051). Effect sizes for these comparisons were small (partial eta-squared < 0.06).

**Effect of age.** A significant age-dependent relationship was established for mean error values when performing spirals of type 2 (*p* = 0.047), type 3 (*p* = 0.044), and type 4 (*p* = 0.008), as well as for mean stylus press frequency when drawing spiral type 3 (*p* = 0.017) and mean velocity for spiral type 4 (*p* = 0.023). These results indicate that with increasing age, task accuracy predictably declines, and this effect is most pronounced for more complex spiral types (with the starting point outside).

**Figure 2:**
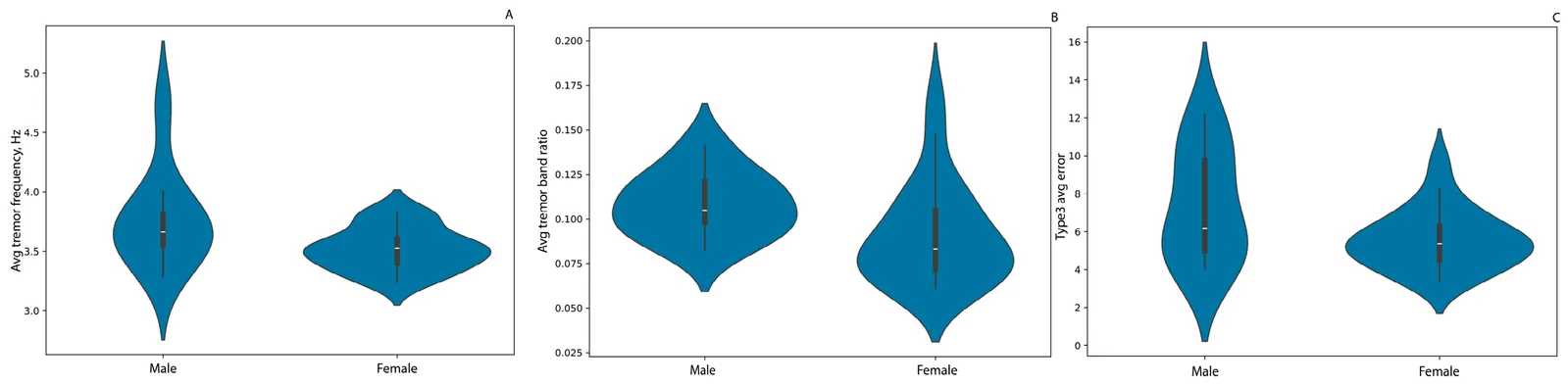
Distribution of values of dominant tremor frequency (**A**), ratio of tremor frequency power to total power (**B**) and mean error for spiral type 3 (**C**) by participant sex (male/female).

**Effect of sex.** The generalized model revealed a significant effect of sex (*p* = 0.032) on the following DAST parameters: total spiral area (*p* = 0.050), dominant tremor frequency (*p* = 0.007), ratio of tremor frequency power to total power (*p* = 0.005), mean velocity for spiral type 1 (*p* = 0.048), velocity variability for spiral type 1 (*p* = 0.046), as well as mean error for spiral type 3 (*p* = 0.023) and type 4 (*p* = 0.039). Figure 2 presents the distributions of 3 measures most closely associated with sex: dominant tremor frequency, ratio of tremor power to total power, and mean error for spiral type 3. Since the CG consisted exclusively of women, sex and group were partly collinear, and the sex effect cannot be fully disentangled from the group effect. We therefore report these findings descriptively rather than as an independent effect of sex.

**Effect of study stage.** Analysis showed a significant effect of study stage on the mean number of stylus presses (*p* = 0.011), reflecting a possible influence of a learning effect or adaptation to task performance upon repeated testing.

### 3.2. Between-Group Differences in DAST Parameters Across Study Stages

At the first stage (baseline, before the iTBS course), statistically significant differences between the ExpG and CG were revealed for a wide range of DAST parameters: trial duration (*p* = 0.0329), total path length (*p* = 0.0196), total error (*p* = 0.0020), median velocity (*p* = 0.0255), velocity variability (*p* = 0.0112), ratio of tremor frequency power to total power (*p* = 0.0229), coefficient of variation in velocity (*p* = 0.0112), length-normalized error (*p* = 0.0017), as well as a number of measures for individual spiral types, including mean velocity and mean duration for spiral types 1 and 4 (*p* = 0.0149–0.0473). The most pronounced differences were observed for spatial parameters reflecting task accuracy. Effect sizes for these between-group differences were small to moderate (rank-biserial correlation 0.20–0.50).

At the second stage (1.5 months after the first visit), the number of significant between-group differences decreased: differences remained for total error (*p* = 0.0064), the ratio of tremor frequency power to total power (*p* = 0.0219), length-normalized error (*p* = 0.0055), as well as for mean duration for spiral type 1 (*p* = 0.0399) and mean stylus press frequency for spiral type 2 (*p* = 0.0385).

At the third stage (6 months after the first visit), statistically significant between-group differences persisted exclusively in two spatial parameters: total error (*p* = 0.0251) and length-normalized error (*p* = 0.0123). These measures, reflecting spatial movement accuracy and coordination, remained significant throughout the entire observation period, in contrast to temporal and kinematic parameters, for which differences were no longer evident by the second stage.

**Figure 3:**
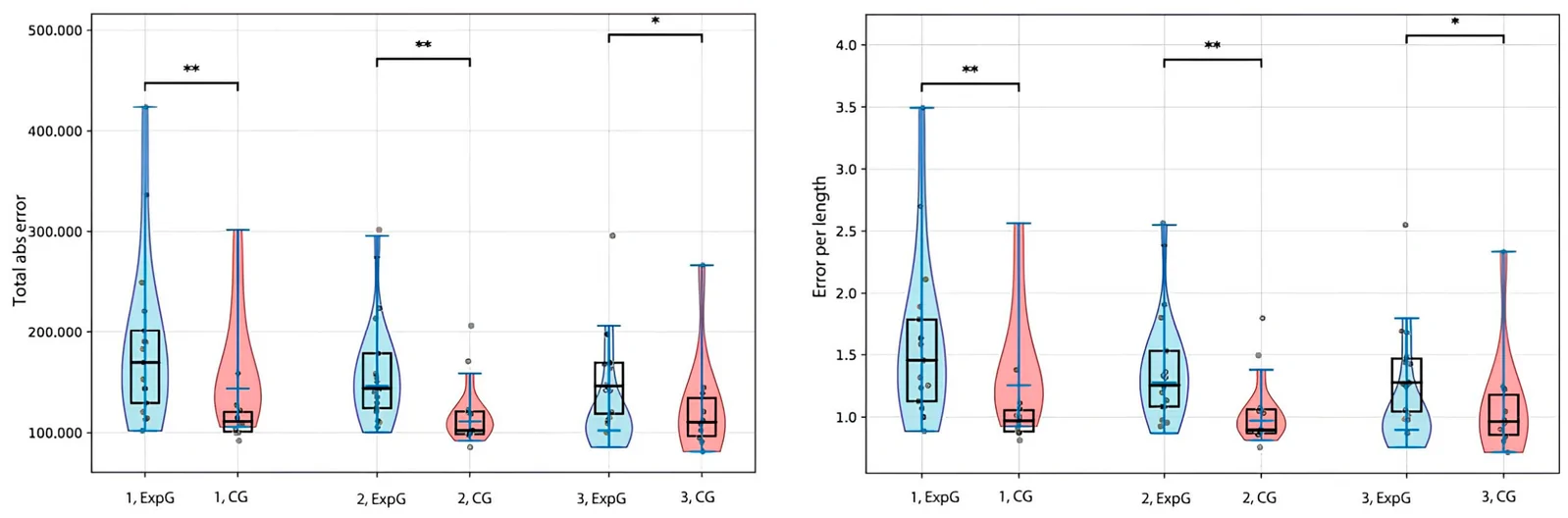
Comparison of the ExpG and CG at stages 1, 2, and 3 for total error and length-normalized error. Data are presented as mean ± standard error of the mean. Note: ExpG—experimental group; CG—control group; * *p* < 0.05; ** *p* < 0.01.

Figure 3 presents the dynamics of total error and length-normalized error for the ExpG and CG across the three study stages. It can be seen that between-group differences for these measures persist at all time points, although absolute values tend to decrease somewhat in the ExpG.

### 3.3. Longitudinal Changes in DAST Parameters Following TMS

**Figure 4:**
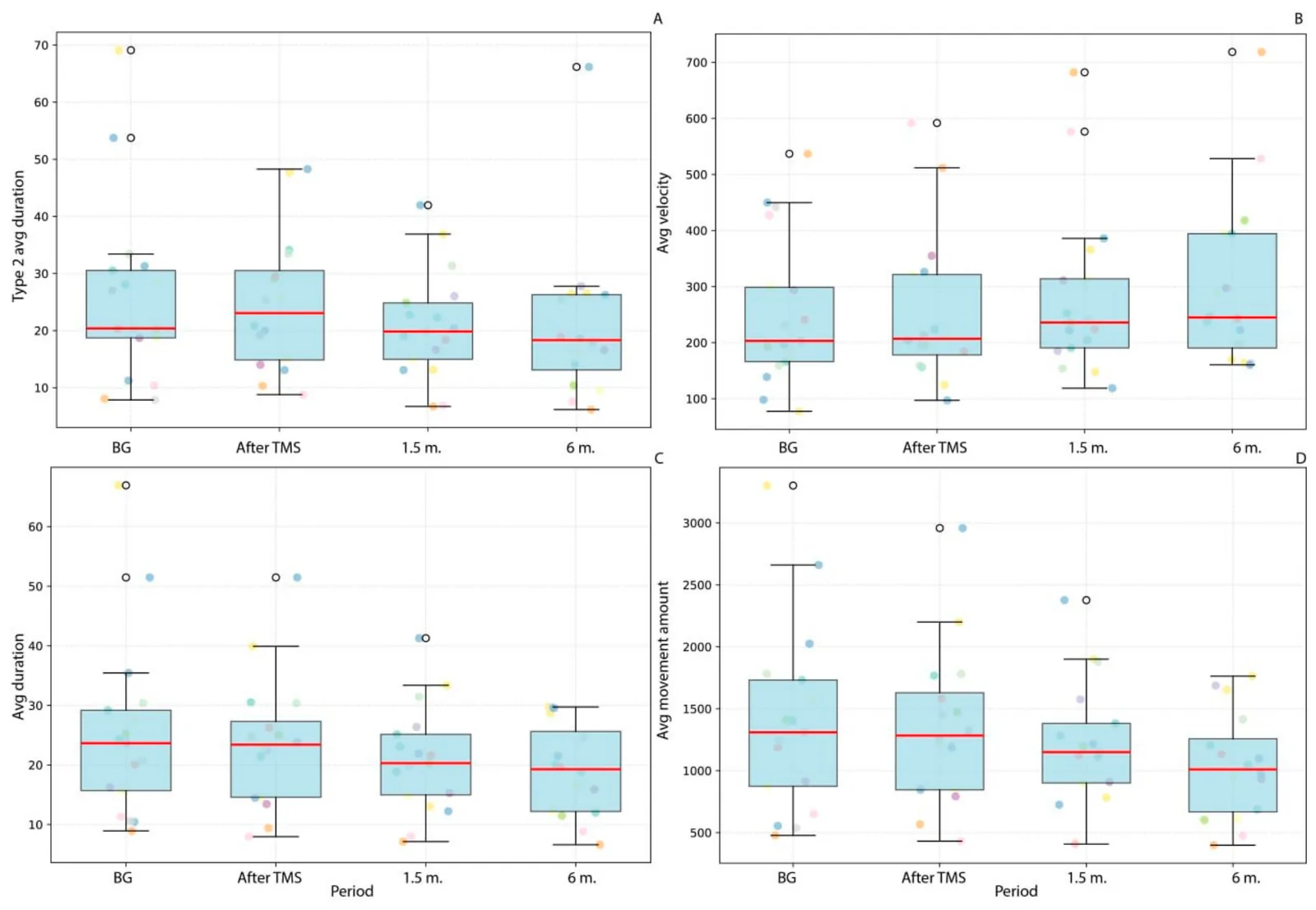
Dynamics of DAST parameter changes in the experimental group: (**A**)—mean duration for spiral type 2; (**B**)—mean task velocity; (**C**)—overall trial duration; (**D**)—mean number of movements. Note: The time points are BG—before the iTBS course; After TMS—immediately after the iTBS course, 1.5 m—at 1.5 months; 6 m—at 6 months.

Within-group analysis of the ExpG across four time points (baseline, immediately after the iTBS course, at 1.5 months, and at 6 months) revealed statistically significant dynamics for the following measures: mean number of movements (*p* = 0.0485), trial duration (*p* = 0.0437), mean velocity (*p* = 0.0437), and mean duration for spiral type 2 (*p* = 0.0029). Effect sizes were small to moderate (Kendall’s W 0.10–0.30). Bonferroni-corrected post-hoc comparisons did not reach the significance threshold (*p*_corr = 0.0644). Figure 4A–D presents the dynamics of each of these parameters.

Post-hoc pairwise comparisons revealed a nonsignificant difference between the state immediately after the iTBS course and at 1.5 months for mean number of movements (*p*_corr = 0.0644), as well as between the baseline visit and 1.5 months for mean duration of spiral type 2 (*p*_corr = 0.0644). Although the corrected *p*-values did not reach the formal significance level (α = 0.05), the direction of the observed trends was compatible with a possible sustained change beyond the immediate post-treatment period. However, these comparisons did not reach statistical significance after multiplicity correction and, in the absence of a sham-treated PD group, cannot be regarded as confirmatory evidence; therefore, persistence of the change at 1.5 months remains uncertain. It is noteworthy that the direction of changes for all 4 parameters was consistent: an increase in mean velocity and a decrease in duration and number of movements. This pattern is compatible with improved task performance efficiency, although it cannot be attributed to iTBS specifically.

Additionally, we report that MDS-UPDRS III scores did not show a significant change following the iTBS course or at subsequent follow-up stages. Discomfort and alertness ratings remained stable across sessions, with no adverse events reported. No participant withdrew from the study, and all patients completed the full course of 10 iTBS sessions and all scheduled follow-up assessments.

### 3.4. DAST Parameters Stratified by Hoehn and Yahr Stage

Stratification of ExpG patients by PD stage according to the Hoehn and Yahr scale (HY-2, *n* = 8, and HY-3, *n* = 9) revealed a limited number of statistically significant differences in DAST parameters. At the first stage (baseline), differences between the subgroups were recorded for the mean velocity of spiral type 2 (*p* = 0.0176), reflecting more pronounced bradykinesia in patients with HY-3. In the post-intervention period (after the iTBS course), significant differences were established for mean stylus press frequency when drawing spiral types 1 (*p* = 0.0384) and 4 (*p* = 0.0283), which may indicate different dynamic adaptation of the motor pattern depending on disease severity. At the second stage (1.5 months), differences persisted for the coefficient of variation in error (*p* = 0.0205), with HY-3 patients demonstrating higher error variability, reflecting lower task stability. At the third stage (6 months), no statistically significant differences between the subgroups were found; only trends toward differences were noted for velocity variability for spiral type 2 (*p* = 0.0496), standard deviation of velocity (*p* = 0.0691), mean velocity error (*p* = 0.0879), and mean stylus press frequency for spiral types 1 (*p* = 0.0746) and 4 (*p* = 0.0855). These are unadjusted *p*-values; the between-stage comparisons were exploratory and were not corrected for multiplicity, and the nominal value of *p* = 0.0496 should therefore be interpreted with caution. Thus, differences between HY-2 and HY-3 stages in DAST parameters were minimal and inconsistent. Given the small subgroup sizes, this study did not provide convincing evidence of stage discrimination rather than demonstrating an absence of discriminative ability; the precision of these comparisons is limited.

## 4. Discussion

The study revealed modest within-group changes in several DAST parameters, including increased mean velocity, reduced trial duration, and decreased number of movements, although these changes were close to the significance threshold and did not survive correction in post-hoc comparisons. At the same time, spatial measures such as total error and length-normalized error maintained stable between-group differences between PD patients and healthy subjects throughout the entire observation period, without showing significant improvement following stimulation. A moderate dependence of several DAST parameters on age and sex was revealed, as were minimal differences between HY-2 and HY-3 stages, indicating differential sensitivity of different classes of measures to therapeutic intervention and the limited ability of the test to distinguish adjacent stages of the disease. These preliminary findings support the potential of DAST as a candidate instrument for detecting motor changes at the level of measurement sensitivity in neurodegenerative disorders; however, its responsiveness to neuromodulatory interventions requires confirmation in larger studies, including those using a sham intervention [31,32,33].

### 4.1. Differential Sensitivity of Kinematic and Spatial DAST Parameters to DLPFC Neuromodulation

The changes in temporal and kinematic DAST parameters observed in the present study are consistent with findings from studies in which high-frequency TMS of the DLPFC facilitated an increase in maximum velocity during goal-directed movements, associated with modulation of visuospatial information processing and motor planning [34]. However, in the absence of a sham-controlled PD group, these changes cannot be attributed to iTBS with certainty. This effect appears physiologically plausible, as the DLPFC, through its functional connections with the dorsal visual stream, is involved in sensorimotor integration and cognitive motor control. Dopamine deficiency in the ventral tegmental area in PD leads to impaired signal transmission to the DLPFC, which can alter the temporal and kinematic characteristics of motor tasks. In this context, the positive effect of DLPFC stimulation on DAST parameters may be related to restoration of prefrontal mechanisms of motor control [35,36].

The observed changes in temporal DAST measures following the iTBS DLPFC course are broadly consistent with data obtained from assessing other motor functions. A meta-analysis summarizing the results of randomized controlled trials demonstrated that repetitive TMS exerts a small to moderate positive effect on overall motor function as measured by the UPDRS, as well as on functional mobility [37]. Moreover, DLPFC stimulation may exert a specific influence on cognitive motor control and strategies for performing graphomotor tasks, making DAST a particularly sensitive tool for detecting such changes. The neurophysiological basis of the observed effects is likely the capacity of rTMS to modulate dopamine release in the caudate nucleus and putamen, as well as in their cortico-striatal projections [38,39,40]. Furthermore, theta-burst stimulation, as applied in the present study, offers additional advantages—short session duration and low stimulation intensity—and its application over the DLPFC has demonstrated therapeutic effects on depression in PD patients [25,41]. It has also been established that rTMS exerts a stimulatory influence on neuroplasticity processes, including mechanisms of long-term potentiation and depression, neuronal sprouting, and regulation of gene expression associated with brain-derived neurotrophic factor synthesis [42,43,44,45]. This may provide a basis for slowing neurodegenerative and cognitive decline, thereby potentially mediating the long-term effects of stimulation.

In contrast to temporal and kinematic parameters, spatial DAST measures (total error and length-normalized error) did not demonstrate significant change over the study period and maintained stable between-group differences at all observation stages. This pattern may be consistent with a greater involvement of prefrontal cognitive-motor control in the parameters that changed, and of other motor circuits in those that did not; however, the present behavioral study cannot directly establish the underlying neural mechanism. The direct execution of movements and parameters of their spatial accuracy are more closely associated with the functioning of the primary motor cortex, cerebellum and basal ganglia; however, this interpretation remains speculative [8,46]. Moreover, the effects of iTBS over the DLPFC are generally modulatory and indirect in nature, mediated through frontostriatal networks, and more often manifest as changes in cognitive aspects of motor behavior (e.g., task execution strategies, velocity, movement variability) rather than direct improvement in spatial accuracy or tremor suppression [47]. It should also be considered that spatial DAST parameters exhibit differential sensitivity: measures based on deviation from the reference trajectory are relatively stable and less sensitive to short-term neuromodulatory effects compared to temporal and kinematic movement characteristics. The clinical significance of DLPFC stimulation is supported by evidence of improvement in UPDRS III scores and reduction in Timed Up and Go test performance with high-frequency TMS, with similar effects observed also during stimulation of the supplementary motor area and primary motor cortex [48,49].

### 4.2. Demographic and Disease-Severity Influences on DAST Parameters

Age-related effects on DAST parameters are consistent with a population-based study of nearly two thousand participants aged 45 years and older, showing that age is associated with deterioration in fine motor skills as assessed by both clinical evaluation and quantitative spiral test analysis [50]. Additional confirmation comes from findings that older participants exhibited higher press force and longer reaction times during spiral tracing; although stylus use improved performance compared to finger tracing, these age-related increases persisted [51]. Age-associated neuronal changes manifest behaviorally as slowed reaction time, reduced motor control, and impaired coordination, and are linked to excessive activation of motor and prefrontal regions; recruitment of additional ipsilateral motor areas may serve a compensatory function but requires greater neural resources and may slow motor responses [52,53].

Sex effects in the present study may reflect the all-female CG (we report these findings descriptively, since they cannot be attributed to sex independently of group membership). Literature data remain equivocal: some studies found no significant sex differences in spiral test performance [11], whereas a population-based study of 1282 healthy subjects showed higher spiral test scores in males and in the non-dominant hand [15]. Further investigation of sex- and hemisphere-related effects is warranted, particularly in samples with unbalanced gender composition [54].

PD-related DAST alterations are corroborated by a large study of 138 PD patients (50 early-stage) and 150 healthy subjects, in which spiral indices reflecting execution speed, mean drawing velocity, density, smoothness, and loop variability differed significantly between groups; PD patients took longer to complete the task, and their spirals were more compact and more variable in shape [11]. In another study using an optoelectronic system, PD patients in the “on” state showed spatial organization, size, and regularity comparable to controls, although drawing velocity was reduced [55]. Similarly, PD patients drew spirals more slowly on a digital tablet, with micrographia features compared to healthy subjects [56]. Thus, spiral drawings in PD are characterized by reduced execution speed, decreased size, and increased force variability; additional features include kinematic spectral peaks predominantly in the 4–7 Hz range, reflecting tremor contribution [57,58]. Reduced DAST speed is attributed to bradykinesia and impaired sensorimotor integration; decreased movement amplitude and pen pressure variability are considered compensatory-adaptive strategies that reduce the impact of sensorimotor disturbances, including tremor, on movement accuracy and stability [59,60,61].

Weak stage-related DAST differences are consistent with previous findings: despite reductions in spiral drawing speed and pen pressure with increasing disease severity, these parameters individually possess limited diagnostic value for differentiating disease stages. Correlation of speed and pen pressure with clinical severity was moderate (Spearman’s rho = 0.4), indicating informativeness for comparing PD patients with healthy individuals but not for reliable discrimination of clinical stages. A composite index of speed and pen pressure (CISP) showed a stronger association with PD severity than its constituent parameters; however, even this measure did not reliably differentiate adjacent stages (1st vs. 2nd, and 2nd vs. 3rd on the HY scale) [9,62]. Other indices, such as first-order zero crossing, second-order smoothness, and mean velocity, correlated most strongly with total UPDRS III and showed a gradient change with increasing motor symptom severity [63]. Consequently, the diagnostic significance of the spiral drawing test likely depends on the selected measures, calculation methods, and sample characteristics [64,65].

It should be noted that the present sample, particularly the CG, was relatively small, which may have limited the ability to detect small but potentially meaningful between-group differences. This consideration is relevant when interpreting the DAST parameters, as the CG served as the reference for normal performance. Given the limited precision of this feasibility sample, the absence of significant differences for some spatial and temporal metrics should be interpreted with caution, rather than as evidence of a true absence of effect. Accordingly, future studies with larger and more balanced groups would be valuable to confirm the present findings, provide more precise estimates of the effects of iTBS on DAST parameters, and enable subgroup analyses stratified by disease stage and sex.

Thus, the obtained data suggest a possible differential responsiveness of the kinematic, temporal and spatial parameters of DAST in this exploratory study, which may reflect differences in the neurophysiological mechanisms underlying these classes of measures. The stability of between-group differences in spatial metrics and the identified influence of demographic factors underscore the need to account for these factors when interpreting results and to standardize protocols for the clinical application of DAST. In a broader context, the use of NIBS in PD continues to attract research attention, particularly regarding its influence on cognitive-motor integration, which highlights the relevance of further investigation into the combined application of neurophysiological interventions and graphomotor assessment methods. Furthermore, modern approaches to data processing, including deep learning methods and advanced diagnostic techniques, open up new prospects for automated analysis of recorded parameters and multimodal biomarker identification, which can significantly expand the evaluative and prognostic capabilities of the test in research settings and, potentially, in clinical practice [66,67,68,69].

## 5. Conclusions

In this exploratory feasibility study, within-group changes were observed in temporal and kinematic DAST parameters, whereas spatial measures showed no significant change and maintained stable between-group differences throughout the observation period. It may be tentatively hypothesized that DLPFC neurostimulation primarily influences motor planning processes and movement execution strategies, whereas the basic mechanisms of trajectory implementation and spatial coordination are mediated by other cortico-subcortical structures (this interpretation is presented as a hypothesis, since the present behavioral study cannot directly establish the underlying neural mechanism). The revealed dependence of several DAST parameters on age and sex confirms the need to account for demographic factors when interpreting the results, while the minimal differences between HY-2 and HY-3 stages indicate the limited ability of the test to differentiate adjacent stages of the disease.

The obtained results support the use of DAST as an additional objective tool for detecting motor changes in PD at the instrument level, while its responsiveness to neuromodulatory interventions and clinical utility remain to be established. From a clinical perspective, the DAST may be useful for longitudinal monitoring of motor parameters during therapy; however, its use for optimizing stimulation protocols based on quantitative feedback is not supported by the present data. Further studies involving larger and more balanced samples, along with standardization of data collection and analysis procedures, will allow clarification of the clinical significance of individual DAST parameters and the determination of their place in the comprehensive assessment of motor impairments in neurodegenerative diseases.

## 6. Study Limitations

When interpreting the obtained results, a number of methodological limitations should be taken into account. First, the relatively small sample size, particularly in the CG, limits the statistical power of the study. The sample should be regarded as a pilot cohort without prospective power justification, and the influence of random variations on the revealed between-group differences cannot be completely excluded. Specifically, given the limited precision of this feasibility sample, smaller but clinically relevant differences may have been missed. Moreover, the CG consisted exclusively of women, whereas the sex ratio in the ExpG was balanced. This makes sex and group partly collinear; adding sex as a covariate does not resolve this collinearity, and the reported sex effects should therefore be regarded as descriptive rather than independent. Despite this limitation, we considered it preferable to report the available data with appropriate caveats rather than omit these observations entirely, since they may inform the design of future studies with balanced groups. The assessment of PD patients was performed exclusively in the “on” state, and the ExpG did not include a sham-controlled arm. Sham TMS was administered only to the CG. Consequently, the observed within-group changes cannot be clearly distinguished from practice or learning effects, regression to the mean, or medication fluctuation. These factors limit the attribution of the observed changes to iTBS and restrict extrapolation of the results to patients in the “off” state. The DAST, despite its high sensitivity and objectivity compared to clinical scales, is not devoid of relative variability. Factors contributing to this include individual differences in interpretation of instructions, possible learning effects upon repeated performance, as well as technical characteristics of the equipment used (sampling rate, accuracy of pressure and coordinate registration). Although the testing procedure was standardized for all participants, these factors could have introduced additional variability into the measured parameters. Our study did not include objective assessment of the effect of TMS on non-motor symptoms, including cognitive functions, affective disorders, or quality of life, which does not allow for conclusions to be drawn regarding the breadth of the therapeutic effect of DLPFC stimulation and its relationship with changes in graphomotor parameters. Also, all participants in the study were right-handed, which limits the generalizability of the obtained results to left-handed patients. Furthermore, the absence of long-term follow-up beyond 6 months does not allow assessment of the stability of the registered effects and the possibility of their clinically significant preservation in the long term. The linear mixed model was limited to the baseline, 1.5-month, and 6-month time points, as the immediate post-treatment measurement was available only in the PD group; the model therefore does not capture the immediate post-iTBS period. Given the exploratory design and the number of metrics analyzed, the results should be regarded as hypothesis-generating and require confirmation in larger studies. Overall, the limitations listed affect the causal interpretation of the findings to varying degrees. These considerations should be taken into account when planning future studies aimed at further validation of DAST as a tool for monitoring motor changes in PD.

## Figures and Tables

**Table 1:** Demographic and clinical characteristics of the study participants.

Parameter	ExpG, *n* = 17	CG, *n* = 10	*p*-Value
Age, years (M ± SD)	64.61 ± 6.34	66.10 ± 3.45	0.507
Sex (M/F)	8/9	0/10	0.025
Dominant hand (right/left)	17/0	10/0	1000
Clinical profile (M ± SD)—only for ExpG: HY stage/disease duration, years/MDS-UPDRS III	2.82 ± 0.64 (2–4)/6.7 ± 4.44/17.59 ± 7.69		

Note: M—mean, SD—standard deviation, HY—Hoehn and Yahr. The groups were comparable in age (*p* = 0.507); the CG comprised only women, which is a limitation accounted for as a covariate.

## Data Availability

The data that support the findings of this study are available on request from the corresponding author (due to privacy, legal, and ethical reasons).
